# From benchmark to user: an end-to-end evaluation audit of an AI-based oral health screening prototype

**DOI:** 10.3389/fdata.2026.1972023

**Published:** 2026-10-28

**Authors:** Ivan Cheng Jun Char, Abdullah Sayed Ahmed Sayed, Kazuki Ichikawa, Youssif Mahmoud Gomaa Sayed, Abdurrahman Bin Milhan, Doreen Ying Ying Sim, Anand Ramanathan

**Affiliations:** 1 School of Computer and Mathematical Sciences, University of Nottingham Malaysia, Semenyih, Selangor, Malaysia; 2 Department of Oral and Maxillofacial Clinical Sciences, Faculty of Dentistry, Universiti Malaya, Kuala Lumpur, Malaysia; 3 Oral Cancer Research and Coordinating Centre, Faculty of Dentistry, Universiti Malaya, Kuala Lumpur, Malaysia

**Keywords:** annotation quality, benchmark contamination, data leakage, digital public health, end-to-end validation, machine learning evaluation, oral health screening, reproducibility

## Abstract

**Background:**

Artificial intelligence (AI) for oral screening is usually judged by aggregate detection metrics on public benchmarks whose partitions, labels, and relevance to users are rarely checked.

**Methods:**

We audited a prototype combining a YOLOv8m detector for six conditions (1,500 test images), a DenseNet169 histopathology network, and a ResNet18 router. Under a pre-specified plan, we measured duplication across partitions, tested removal of duplicated test images against 10,000 matched controls, compared duplicated copies' labels and predictions, compared the library versions, and ran every test image through the application pipeline.

**Results:**

Near-duplicates of training images accounted for 17.3% of test and 17.1% of validation images (lower bounds). Removing duplicated test images changed mean average precision (mAP)@0.5 by +0.001 (95% confidence interval [CI] −0.012 to +0.014). Only 22% of duplicated photographs had identical labels on both copies; exploratory analysis suggested memorization of training labels in orientations seen in training. A library update moved mAP@0.5:0.95 by −0.012 with identical predictions. The router rejected 23% of in-domain photographs and 51% of photographs with ulcers, so ulcer sensitivity per image was 0.36 despite the highest benchmark recall (0.91). A class-mapping defect, still live in a public demonstration in September 2026, gave the wrong name to 73% of findings. The histopathology classifier classified every image as positive; its label was not a diagnosis.

**Conclusion:**

Duplication compromised the benchmark's independence without detectably changing aggregate mAP; the defects that changed what was reported or displayed lay in the labels, evaluation software, and pipeline. Evaluations should verify partitions and labels, pin the library, and test deployed systems end to end.

## 1 Introduction

Oral diseases affect an estimated 3.7 billion people worldwide and are among the most prevalent non-communicable conditions (World Health Organization, 2025). Dental caries and periodontal disease are largely preventable through early intervention, yet both are concentrated in populations with the least access to routine dental examination (World Health Organization, 2025). This gap motivates consumer-facing tools that apply computer vision to smartphone photographs of the oral cavity (Ramírez-Pedraza et al., 2025).

Evaluation of these tools has been inherited from computer vision. Performance is reported as mean average precision (mAP), aggregate precision and recall, or aggregate accuracy, measures designed to rank architectures on benchmark datasets rather than to characterize a screening instrument used by the public. Reporting guidelines for clinical prediction and medical imaging require considerably more (Mongan et al., 2020; Collins et al., 2024; Tejani et al., 2024; Schwendicke et al., 2021), and reviews of medical imaging AI have documented how often benchmark results fail to translate into practice (Roberts et al., 2021; Varoquaux and Cheplygina, 2022; Kelly et al., 2019; Nagendran et al., 2020).

Three assumptions sit beneath a benchmark figure. The first is that test images are independent of training images; where public datasets are assembled from several sources and split at the file level, near-duplicates can cross partitions unnoticed (Roberts et al., 2021; Barz and Denzler, 2020; Kapoor and Narayanan, 2023; Bussola et al., 2021). The second is that the labels are valid. The third is that the model's metrics describe what the deployed system tells its user. Each is usually assumed rather than checked, and when leakage is found, it is commonly taken to mean that performance was overstated, an inference that is itself empirical.

We report an audit of a three-model oral screening prototype that tests these assumptions at three levels: the model, the pipeline around it, and the output a user receives. Our aim is not to propose an architecture but to establish, for one system whose code, weights, and data are openly available, which evaluation claims survive verification.

## 2 Methods

### 2.1 System and data

The prototype (Figure 1A) comprises a ResNet18 router (He et al., 2016) that sends an uploaded image to a clinical or histopathology pathway, a YOLOv8m detector (Jocher et al., 2023) for six visible conditions, and a DenseNet169 multi-task network (Huang et al., 2017) for three histopathological features (mitotic figures, multiple nucleoli, and nuclear hyperchromatism), and is served through a Python web application that was also publicly hosted as a demonstration (Section 3.6).

**Figure 1:**
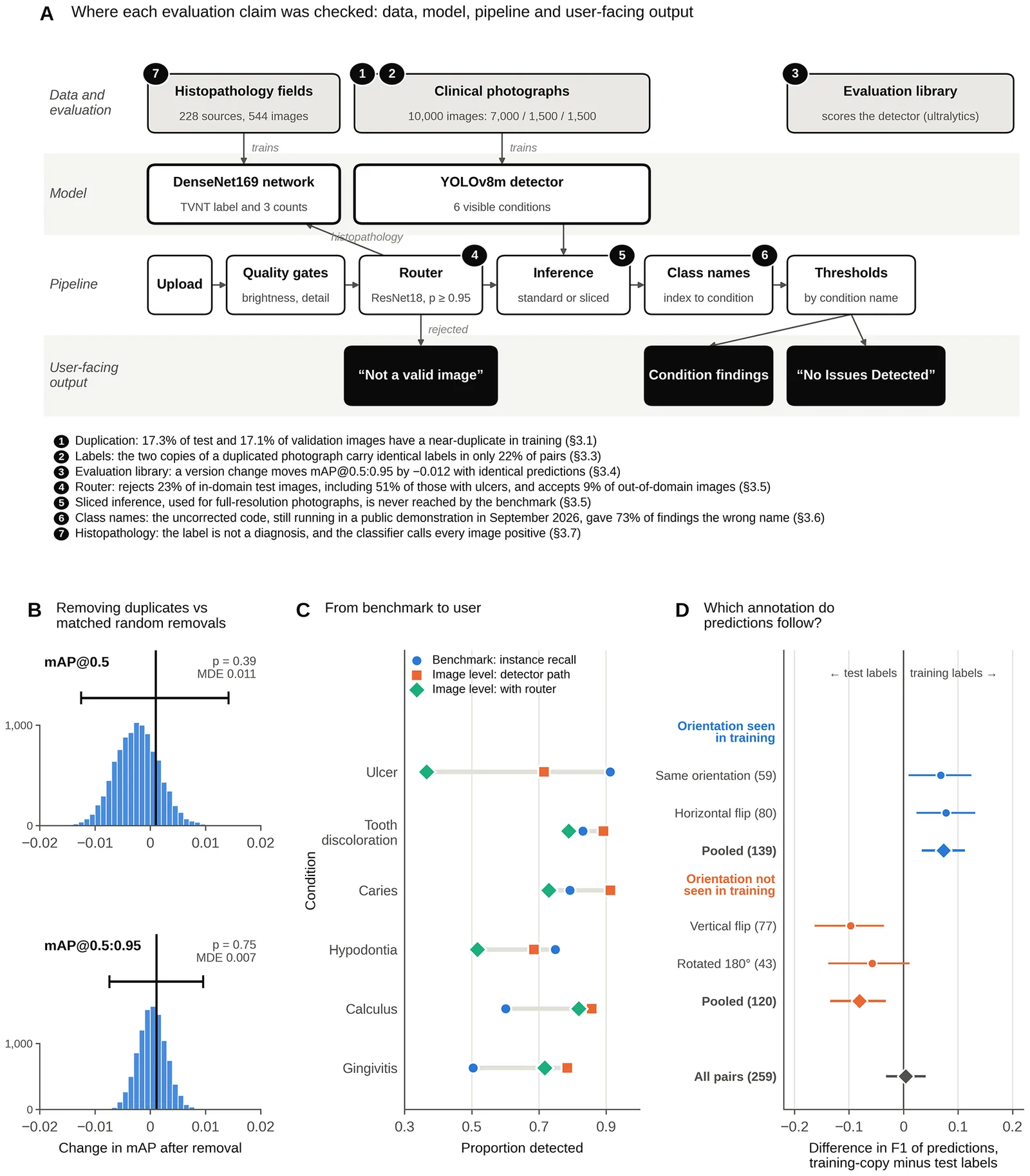
Evaluation audit of the oral screening prototype. **(A)** System components arranged by layer (data and evaluation software, model, pipeline, and user-facing output), with the points at which evaluation claims were checked; numbers refer to the key and to the Results sections. **(B)** Change in mAP@0.5 **(top)** and mAP@0.5:0.95 (bottom) when the 259 duplicated test images are removed (vertical line), with its 95% cluster-bootstrap interval (bracket), against 10,000 control sets that remove 259 non-duplicated images matched on dominant class and instance count (histogram); *p*, randomization *p*-value; MDE, minimum detectable effect. **(C)** Detection of each condition at three levels: instance-level recall on the benchmark at the library's operating point (circles), the proportion of images annotated with the condition in which the application's detector reported it (squares), and the same when the router is in place (diamonds). **(D)** For the 259 duplicated test images, the difference in box-level F1 when the detector's predictions on the test image are scored against the training copy's annotation rather than the test image's own (positive values: closer to the training copy's labels), according to the transform relating the two copies (number of pairs), with 95% cluster-bootstrap intervals. The training augmentation included horizontal flips but no vertical flips or rotations, so the paired training copy could have been seen in the test image's orientation only if the two were identical or left–right mirror images. Exploratory analysis. Detector metrics in **(B,C)**: Ultralytics 8.3.231, CPU, batch size 1.

Both datasets are public projects on Roboflow Universe in the authors' workspace, licensed under CC BY 4.0. Clinical photographs came from version 1 of the first dataset [SEGP (University of Nottingham Malaysia), 2025]: 10,000 images annotated with bounding boxes for six conditions, partitioned by the platform into 7,000 training, 1,500 validation, and 1,500 test images. The platform applied no augmentation and records no upstream source for this project, and its filenames carry no patient or source identifiers. Histopathology images came from version 2 of the second dataset [SEGP (University of Nottingham Malaysia), 2026], which has the same name, image count, and partition sizes as an earlier public project (Oral Cancer, 2023): 228 source images expanded to 544 by three augmented versions of each training image (474/44/26). Every histopathology filename follows the pattern oral squamous cell carcinoma (OSCC)_400x_*n*, the naming convention used by public copies of a histopathology database (Rahman et al., 2020) used for its 400 × fields from OSCC; the project itself does not state a source.

### 2.2 Labels and training records

Clinical annotations were used as supplied, without review by a dentist. The histopathology project contains bounding boxes for the three nuclear features and no diagnostic label. The training pipeline derived a count target per feature and a binary tumor-vs.-non-tumor (TVNT) target, positive whenever an image contained any box (526 positive, 18 negative). The network (ImageNet initialization, four output heads, Adam at 1 × 10^−4^, 50 epochs, seed 42) was trained after all three platform partitions were pooled and re-split at the image level (435/109).

The detector's training is described in the metadata stored in the released checkpoint (Supplementary Table S8.1), which supersedes our original description. The checkpoint was written on 12 December 2025 by Ultralytics 8.3.231 on a single GPU in an unrecorded software environment, with a batch size of 100, a limit of 200 epochs, and early stopping patience of 20. Training ran for 48 epochs, and the retained weights are from epoch 28, which had the highest validation mAP@0.5:0.95, the library's selection criterion. The checkpoint records seed 0 and deterministic mode, and no other run of this configuration is recorded. Its hyperparameters differ from the library defaults, and its run name suggests that they came from the library's evolutionary tuning, which also scores candidates on the validation partition; no record of that search was kept. Training augmentation included horizontal flips (probability 0.28) but no vertical flips or rotations. The run was initialized from an earlier checkpoint (6 December 2025; 50 epochs, library defaults) trained on a dataset directory of the same name, and that checkpoint was itself initialized from a third checkpoint that was not retained.

### 2.3 Audit design and evaluator

The audit began in August 2026 as an exploratory study; Supplementary material S6 gives its chronology, including analyses revised after initial results. For this revision, the planned analyses were specified in a plan committed to the repository before any of them were run (commit 7ea8df2, 21 September 2026). Analyses added after the results are labeled exploratory, and deviations from the plan are listed in Supplementary Table S8.8. No model was retrained; all detector results derive from the released checkpoint (SHA-256 4de8714f…7aad0cc). The audit code was written and run with the assistance of a generative AI model (Claude Opus 5, Anthropic) under the authors' direction (see the Generative AI statement).

Except where a library version is compared (Section 3.4) or the training log is cited (Sections 2.2 and 4.1), all detector metrics come from one evaluator: Ultralytics 8.3.231, the version used to write the checkpoint, on CPU in 32-bit floating point at batch size 1 (image size 640, confidence 0.001, non-maximum suppression intersection over union (IoU) 0.7, at most 300 detections). Subset metrics were computed with the library's own metric function from per-image match statistics cached from its validator, which reproduced its validation exactly for the full split, the de-duplicated split, and the two random splits. At batch size 16, the same version reproduces the values reported during development to within 0.001; batch size 1, which makes letterbox padding independent of batch composition, changes per-class recall by up to 0.009 and mAP by less than 0.0001 (Supplementary Table S8.6).

### 2.4 Duplication and its effect

Each image was reduced to a 32 × 32 greyscale thumbnail, and each test image was compared with every training image by root-mean-square (RMS) difference, taking the minimum over the eight rotations and reflections of the square. Pairs with distances below 6.0, a threshold set in the original audit from the distance distribution before any metric was computed, were flagged; test–training and validation–training pairs were verified at full resolution against random-pair nulls. The same rule was applied to the validation partition and within the test partition. Test images linked by within-test duplication or by a shared training duplicate formed clusters (1,445 clusters for 1,500 images), which served as the resampling unit for all bootstrap intervals on the clinical benchmark.

The primary estimand was the removal effect, Δ = M(test images without a training duplicate) − M(full test split), for M = mAP@0.5 and mAP@0.5:0.95, considered separately. We report a 95% cluster-bootstrap interval (10,000 resamples, stratified by whether a cluster contains a duplicated image; Efron and Tibshirani, 1993) and compare Δ with 10,000 control sets that instead remove the same number of non-duplicated images, stratified by dominant class and number of annotated instances, and with 10,000 unstratified controls. The two-sided randomization *p*-value is the proportion of control sets for which Δ lies at least as far from the control median as the observed Δ. The minimum detectable effect, at 80% power and two-sided α = 0.05, is (1.96 + 0.84) times the standard deviation of the stratified controls. Because no equivalence margin was specified before the original results were seen, we make no equivalence claim (Lakens, 2017). Secondary analyses compared duplicated and non-duplicated images per class, performance across partitions, the annotations of each duplicated test image with those of its training copy (greedy box matching at IoU ≥ 0.5 in the test image's frame), and the recall of predictions on duplicated images against each annotation; box-level F1 between predictions and both annotations was added as an exploratory analysis. We also evaluated the predictions under Ultralytics 8.4.118.

### 2.5 System-level validation

The request path was traced from upload to the displayed text: image-quality gates, the router (softmax threshold 0.95), the detector, the mapping from class index to condition name, per-condition confidence thresholds looked up by that name, and the “No Issues Detected” message. It was replicated offline with the application's own modules, as corrected (Section 3.6), and all 1,500 test images were passed through it both with and without the router. For each condition, we report how often it was reported in images annotated with it and in images not annotated with it, and the outcomes a user would see. The pre-correction path was simulated in full. The router was also evaluated on the histopathology images and on COCO128, a 128-image subset of COCO (Lin et al., 2014) distributed with the detection library, as out-of-domain inputs.

### 2.6 Histopathology metrics

The re-split was reproduced exactly, and the deployed checkpoint was evaluated deterministically. We report the confusion matrix, sensitivity, and specificity with Clopper–Pearson intervals; balanced accuracy; receiver operating characteristic–area under the curve (ROC-AUC) with a bootstrap interval; average precision with each class as target; the Matthews correlation coefficient (MCC); and the base rate (Chicco and Jurman, 2020; Saito and Rehmsmeier, 2015); and for the count heads, mean absolute error (MAE), exact-match rate, and *R*^2^, computed against the mean of the evaluation set.

### 2.7 Ethics

This study used secondary analysis of publicly available, de-identified image collections. No human participants were recruited, and no personal health data were accessed, so ethics approval and informed consent were not required.

## 3 Results

### 3.1 Duplication across all three partitions

Of the 1,500 test images, 259 (17.27%) had a near-duplicate in training, and these carried 23.48% of test instances (Table 1; Supplementary materials S1, S2, S4). Matching at a fixed alignment found 124; the other 135 matched only after reflection or 180° rotation. The validation partition was contaminated at a similar rate, with 256 of 1,500 images (17.07%; Supplementary material S7), all of which were confirmed at full resolution in an exploratory check. Because the platform applied no augmentation, the duplicates were already present in the images when uploaded. These counts are lower bounds. After brightness and contrast were normalized, a further 163 candidate pairs had a full-resolution correlation exceeding 0.95, but the candidates formed a continuum with no natural threshold, and no method used here detects the same lesion photographed twice.

**Table 1:** Duplication in the benchmark and its measured effect on the frozen detector.

Split or statistic	Images	Instances	mAP@0.5	mAP@0.5:0.95
(A) Evaluation sets				
Full test split	1,500	9,688	0.7709	0.4009
Test, without duplicates (ND)	1,241	7,413	0.7719	0.4020
Test, duplicated only (D)	259	2,275	0.6602	0.3151
Training partition	7,000	44,786	0.9342	0.6131
Validation partition	1,500	9,396	0.7577	0.3874
Validation, without its 256 duplicates	1,244	7,179	0.7483	0.3826
(B) Removal effect, Δ = M(ND) – M(full test split)				
Δ (95% cluster-bootstrap CI)			+0.0010 (−0.0125, +0.0142)	+0.0011 (−0.0074, +0.0096)
Stratified controls, mean (SD)			−0.0024 (0.0039)	+0.0003 (0.0025)
Stratified controls, 2.5th to 97.5th percentile			−0.0099 to +0.0053	−0.0046 to +0.0053
Randomization *p*-value, stratified/unstratified			0.39/0.69	0.75/0.62
Percentile of Δ among stratified controls			80.7	62.5
Minimum detectable effect			0.011	0.007
Δ under Ultralytics 8.4.118			+0.0004	+0.0008
(C) Duplicated test images and their training copies (259 pairs)				
Pairs with identical label sets			57 (22.0%)	
Box-level F1, test annotation against training annotation			0.662 (0.623, 0.700)	
mAP@0.5 of the 224 training copies against their own labels			0.882	
Predictions on D at *t* = 0.282, recall against test/training annotation			0.684/0.696	
Exploratory: predictions on D, F1 against test/training annotation			0.680/0.684	
Exploratory: F1 difference, training minus test annotation, all pairs (259)			+0.004 (−0.032, +0.040)	
Orientation seen in training (139 pairs)			+0.074 (+0.033, +0.113)	
Orientation not seen in training (120 pairs)			−0.081 (−0.135, −0.032)	

Metrics: Ultralytics 8.3.231, CPU, batch size 1, except the last row of **(B)**. D, test images with a near-duplicate in training; ND, the remaining test images.

Controls: 10,000 sets each removing 259 non-duplicated test images, stratified by dominant class and instance-count bin (on average 2,139.5 instances removed, against 2,275 for D), and 10,000 unstratified sets. Minimum detectable effect: (1.96 + 0.84) × SD of the stratified controls. Ulcer contributes three instances to D and could not be balanced. Randomization p-value: proportion of control sets whose Δ lies at least as far from the control median as the observed Δ. **(C)** Boxes matched in a class-aware, greedy manner at intersection over union ≥0.5 after mapping the training copy into the test image's frame; F1 values have 95% cluster-bootstrap intervals (10,000 resamples); t, the confidence at which the smoothed mean F1 curve of the full split peaks. Orientation seen in training: the two copies are identical or left–right mirror images, which training augmentation (horizontal flips, but no vertical flips or rotations) could reproduce; not seen: vertical reflection or 180° rotation. Per-class contrasts are in Supplementary Table S8.2 and further concordance analyses in Supplementary Table S8.9. (A), (B) and (C) label the three blocks of the table: the evaluation sets, the removal effect, and the duplicated test images with their training copies.

### 3.2 Removing duplicated test images did not detectably change aggregate mAP

Removing the 259 images changed mAP@0.5 by +0.0010 (95% CI −0.0125 to +0.0142) and mAP@0.5:0.95 by +0.0011 (−0.0074 to +0.0096) (Table 1, Figure 1B). Against control sets that removed 259 non-duplicated images matched on dominant class and instance count, the randomization *p*-value was 0.39 for mAP@0.5 and 0.75 for mAP@0.5:0.95 (unstratified, 0.69 and 0.62). The intervals allow inflation of up to 0.012 in mAP@0.5 and 0.007 in mAP@0.5:0.95; the design had 80% power only for effects of at least 0.011 and 0.007. Of ten per-class recall contrasts, one interval excluded zero, and it favored the non-duplicated images: tooth discoloration at the deployed threshold (−0.067; −0.129 to −0.012), for which precision was also lower on duplicated images at both operating points (Supplementary Table S8.2). Ulcer, with three duplicated instances, could not be compared.

### 3.3 Duplicated test images and their training copies

The detector fitted its training images far more closely than other images (mAP@0.5: 0.934 on training, 0.758 on validation, and 0.771 on test; Table 1). Any advantage from duplication depends on whether the two copies carry the same labels. Only 57 of 259 pairs (22.0%) had identical label sets, and the two annotations of the same photograph agreed with a box-level F1 score of 0.66 (Table 1C). In an exploratory visual check of 24 pairs by the AI agent that ran the audit, every training copy was the same photograph as its test image; the labels differed in 20 cases, most often by a whole condition annotated on one copy only.

Predictions on duplicated test images recovered slightly more of the training copy's boxes than of the test image's own (pre-specified recall of 0.696 against 0.684; exploratory box-level F1 score of 0.684 against 0.680, difference of +0.004, 95% CI of −0.032 to +0.040). Because the recorded training runs used horizontal flips but no vertical flips or rotations (Section 2.2), the detector could have seen the paired training copy in its test image's orientation only when the two were identical or left–right mirror images. In these 139 pairs, predictions agreed better with the training copy's annotation than with the test image's own (+0.074; 0.033 to 0.113). In the other 120 pairs, the difference reversed (−0.081; −0.135 to −0.032; Figure 1D), although agreement with each image's own annotation did not differ between the two groups. The first result is consistent with memorization of training labels; the reversal is unexplained. Both persisted after excluding test images with a second training copy at a fixed alignment, but the grouping was chosen after seeing the per-transform results, and 21 pairs changed groups under our original pairing (Supplementary Table S8.9).

### 3.4 The evaluation library changed the metric without changing a prediction

Evaluating the same cached predictions under Ultralytics 8.4.118 instead of 8.3.231 changed mAP@0.5 by −0.0042 and mAP@0.5:0.95 by −0.0118 on the full split, whereas all 61,159 predictions and their match statistics were bit-identical (Supplementary Table S8.6). An exploratory decomposition located the difference entirely in how average precision is integrated. Version 8.3.231 interpolates precision linearly from the highest recall reached to zero at recall 1.0, crediting the detector with recall it never achieves; version 8.4.118 sets precision to zero beyond the highest recall reached, consistent with the interpolation used in the COCO evaluation (Lin et al., 2014; Padilla et al., 2021). Under 8.4.118, the removal effect was +0.0004 for mAP@0.5 and +0.0008 for mAP@0.5:0.95. Within this experiment, the version change moved each metric by more than the removal of the duplicates did and moved mAP@0.5:0.95 by more than the upper limit of the removal effect's confidence interval.

### 3.5 From benchmark to user

Table 2 and Figure 1C follow each condition from the benchmark to the user-facing output. Instance-level recall ranged from 0.912 for ulcers to 0.504 for gingivitis. At the image level, after applying the application's detector and thresholds, the proportion of images with a condition in which it was reported ranged from 0.685 for hypodontia to 0.912 for caries. For gingivitis, it was 0.785, and no image annotated with gingivitis produced “No Issues Detected,” with or without the router.

**Table 2:** From benchmark to user, by condition, on the 1,500 test images.

Condition	Test instances (images)	Duplicated instances (%)	Deployed threshold	Instance recall	AP@0.5	Reported when present: detector path	Reported when present: with router	Reported when absent: with router
Ulcer	498 (274)	0.60	0.75	0.912	0.957	0.715	0.365	0.000
Tooth discoloration	4,153 (610)	19.87	0.40	0.830	0.878	0.892	0.789	0.083
Caries	1,694 (536)	14.82	0.35	0.792	0.828	0.912	0.729	0.043
Hypodontia	334 (184)	43.11	0.60	0.749	0.768	0.685	0.516	0.001
Calculus	1,465 (342)	31.06	0.25	0.601	0.640	0.857	0.819	0.035
Gingivitis	1,544 (297)	38.67	0.30	0.504	0.554	0.785	0.717	0.037
All conditions	9,688 (1,500)	23.48		0.731	0.771			

Benchmark columns: Ultralytics 8.3.231, CPU, batch size 1, at the instance level, at the library's operating point. Image-level columns: the application pipeline, as corrected, at its per-condition thresholds.

“Reported when present”: the proportion of images annotated with the condition in which the application reported it. “Reported when absent”: the proportion of images not annotated with it in which it was reported; every such image carries another condition, so this is not a false-positive rate in healthy mouths. Outcomes overall: 1,500 images: detector path, at least one finding 92.6% and “No Issues Detected” 7.4%; with the router, at least one finding 73.1%, “No Issues Detected” 3.9%, and rejection as not a valid image 22.9%. Duplicated instances: the share of the condition's test instances that lie in D (Supplementary Material S4). 95% CIs and outcomes per condition are in Supplementary Table S8.3.

The router changed this pattern. With the brightness gate, it rejected 344 of the 1,500 test images (22.9%) and 134 of the 544 histopathology images (24.6%) as not valid oral images. It never confused the clinical and histopathology pathways, but it accepted 11 of 128 COCO images (8.6%; Supplementary Table S8.4). In an exploratory breakdown, rejection depended on the condition, from 6.1% of images with calculus to 51.5% of those with ulcers (Supplementary Table S8.3). With the router in place, image-level sensitivity therefore ranged from 0.365 for ulcers, which had the highest benchmark recall, to 0.819 for calculus; four in five unreported ulcer images were never analyzed. Every test image is under 640 pixels high, so none took the sliced-inference branch (Akyon et al., 2022) that a full-resolution phone photograph would follow. The interface also showed one heading as an internal key and reported router errors as invalid images. Patient-level sensitivity, specificity in healthy mouths, and referral burden cannot be estimated because the benchmark contains no healthy images and no patient identifiers.

### 3.6 The class-mapping defect at the user-facing layer

The deployment layer declared a class order in its first commit that matched no checkpoints in the repository's history. Simulating the pre-correction path on the test split, 1,464 of 1,994 displayed findings (73.4%) carried the wrong condition name, and in 1,074 of 1,500 images (71.6%) every displayed name was wrong; only gingivitis mapped to itself (Supplementary Table S8.5). Because thresholds were looked up using the displayed name, the defect also changed which detections were shown: tooth discoloration, for example, was held to the ulcer threshold of 0.75 rather than 0.40. No model-level metric was affected. In the repository, the mapping is now read from the checkpoint's class list and checked by a regression test. The public demonstration, hosted on Hugging Face Spaces since December 2025 and last updated in April 2026, never received the correction and was still running the pre-correction code until 22 September 2026, when we made it private and took it offline. To our knowledge, it was not used outside the development team.

### 3.7 Histopathology

All 544 images, including all 18 TVNT negatives, are named 400 × OSCC fields. The negatives come from only eight source images, and all four validation negatives have their source image in training. A TVNT negative therefore marks a field designated as carcinoma, in which no feature was annotated, not normal tissue, and the target is not a tumor diagnosis. Independently, the pooled re-split left 96 of 109 validation images (88.1%) with an augmented copy of their source in training (Supplementary material S5), and the checkpoint was selected on that partition.

The classifier assigned the positive class to every image in both partitions (Table 3). A sensitivity of 1.000, accuracy of 0.963 (equal to the base rate), and F1 of 0.981 coexist with specificity of 0.000 (95% CI 0.000–0.602), balanced accuracy of 0.500, and an undefined MCC; on such an imbalanced target, high accuracy and F1 do not indicate useful discrimination. ROC-AUC was 0.831 (0.657–0.971): the scores carry some ranking information, but every score lay above the decision threshold. The AUC in our original submission (0.648) was one realization from a randomly augmented evaluation: the validation subset had inherited the training transform, and five repeated evaluations gave AUCs from 0.605 to 0.788 (Supplementary Table S8.7). Among the count heads, mitotic figures had both the lowest error (MAE 0.135) and the lowest *R*^2^ (0.072). Every validation prediction lay between 0.00 and 0.27 after clipping at zero, so all rounded to zero, and the 93.6% exact-match rate is the proportion of validation images without mitotic figures.

**Table 3:** Histopathology network, deployed checkpoint, deterministic evaluation, on the reproduced validation partition (*n* = 109) unless otherwise stated.

Metric	Mitotic figures	Multiple nucleoli	Nuclear hyperchromatism
(A) Tumor-vs.-non-tumor (TVNT) classifier			
Confusion matrix, TN/FP/FN/TP (training partition)		0/4/0/105 (0/14/0/421)	
Base rate (positive)/accuracy/F1		0.963/0.963/0.981	
Sensitivity (95% CI)		1.000 (0.965–1.000)	
Specificity (95% CI)		0.000 (0.000–0.602)	
Balanced accuracy/MCC		0.500/undefined (0/0)	
ROC-AUC (95% CI)		0.831 (0.657–0.971)	
ROC-AUC as originally reported/range over five augmented repeats		0.648/0.605–0.788	
Average precision, positive/negative class as target		0.992/0.187	
(B) Count heads			
Validation images with the feature present (of 109)	7	71	86
Ground truth, range (mean)	0–2 (0.07)	0–28 (3.91)	0–36 (3.47)
Predictions, range (clipped at 0)	0.00–0.27	0.09–30.44	0.00–27.88
Mean absolute error	0.135	1.753	2.072
Exact-match rate (%)	93.6	18.3	26.6
*R* ^2^	0.072	0.744	0.438

All 544 images are described as 400 × oral squamous cell carcinoma fields; TVNT is positive when any feature box is present. Every image in both partitions scored above the 0.5 decision threshold. The values originally reported came from an evaluation that applied training-time augmentation: MAE 0.144, 2.027, and 2.177; exact-match 93.6%, 12.8%, and 25.7%; and *R*^2^ −0.015, 0.689, and 0.483. *R*^2^ is computed against the mean of the evaluation set. TN, true negatives; FP, false positives; FN, false negatives; TP, true positives. (A), (B) and (C) label the three blocks of the table: the evaluation sets, the removal effect, and the duplicated test images with their training copies.

## 4 Discussion

### 4.1 What the de-duplication experiment shows, and what it does not

The de-duplication experiment asks only whether removing the identified duplicates changes this frozen checkpoint's aggregate metric, and it did not detectably change it. It does not estimate what the model would have scored had it never trained on the duplicates, which would require retraining on de-duplicated, source-level partitions. We did not retrain. A retrained model would be a different model, and the original training cannot be reproduced: its initialization lineage ends in an unretained checkpoint, and neither the hyperparameter search nor the earlier run's partition file was kept. The same gaps limit what can be said about model exposure: we verified duplication between partitions but cannot exclude that an earlier checkpoint in the lineage saw images related to the test set. The audit itself, by contrast, is reproducible: the repository's scripts regenerate its results from pinned data, weights, and environments. The exploratory analysis in Section 3.3 suggests one possible reason why duplication did not detectably inflate this benchmark: where the detector could have seen the paired training copy in the test orientation, its predictions agreed more closely with that copy's annotation, which usually differed from the test annotation. Where duplicates carry consistent labels, the same memorization would be expected to inflate test metrics (Barz and Denzler, 2020; Bussola et al., 2021).

Contamination of the validation partition is not addressed by any filtering of the test split. In the training log, the released checkpoint corresponded to the epoch with the highest validation mAP@0.5:0.95, 0.0011 ahead of the runner-up, and its hyperparameters appear to have been tuned on the same partition. Removing the duplicated validation images lowered that checkpoint's validation mAP@0.5:0.95 by 0.0047 in our evaluator, approximately four times the selection margin; whether this would change the ranking of epochs cannot be determined without per-epoch checkpoints. The histopathology checkpoint was likewise selected on a validation partition in which 88.1% of images had a sibling in training. Correcting model selection requires retraining with independent, source-level partitions.

### 4.2 Provenance, labels, and partitions are separate questions

Dataset provenance, annotation validity, partition independence, and model performance are distinct criteria, and this audit bears on them differently. Because the clinical project records no source and no patient identifiers, independence could only be assessed from image content. Photographs of the same patient, from the same clinical episode, or of the same lesion taken differently cannot be identified; public collections assembled from other collections compound this problem (Roberts et al., 2021). In the histopathology project, the source image of each augmented copy is identifiable, but not the patient; the database from which the images appear to come contains 1,224 images from 230 patients (Rahman et al., 2020). Annotations were not reviewed by a dentist or pathologist, so reported metrics measure agreement with the supplied labels, not diagnostic accuracy. Two annotations of the same photograph were identical in only 22% of pairs and, in those inspected, often differed by a whole condition; therefore, where this can be measured, these annotations show poor reproducibility, which limits how precisely any model can be scored against them. The histopathology target is not valid as a diagnostic target, however consistent its annotations are. Partition independence failed in the clinical project's own partitions and in the histopathology pipeline's re-split. Model performance is conditional on all three.

### 4.3 Levels of validation

Benchmark performance is distinct from internal validation, external validation, clinical validity, and clinical utility (Kelly et al., 2019; Nagendran et al., 2020; Hassanein et al., 2026a). This study reports benchmark performance with internal validation compromised by contamination. There was no external validation, a gap also noted in systematic reviews of dental imaging AI (Hassanein et al., 2026c), and no clinical evaluation or user study, so nothing here supports clinical effectiveness or deployability. Within the system, we separated model validation, pipeline validation, and validation of the user-facing output. The class-mapping defect, the router's rejections, the per-condition thresholds, and the unevaluated sliced-inference branch are invisible to model metrics. The ranking of conditions by benchmark recall was not the ranking a user would experience: instance-level recall understated how often gingivitis, which in its common plaque-induced form is reversible (Chapple et al., 2018), was reported in an image, whereas it overstated performance for ulcers.

### 4.4 Recommendations for reporting

For consumer-facing screening AI, we suggest auditing every partition, including validation, for duplication using content matching that is invariant to reflection and rotation, and reporting the count as a lower bound; checking whether duplicates carry consistent labels; reporting the evaluation library and version beside every detection metric, since versions can differ in how the metric itself is computed (Padilla et al., 2021; Pineau et al., 2021) and evaluation conditions can change the reported performance of an unchanged model (Hassanein et al., 2026b); reporting per-class and image-level results at the deployed operating point, with confusion matrices and metrics that expose performance on the minority class (Chicco and Jurman, 2020; Saito and Rehmsmeier, 2015; Oakden-Rayner et al., 2020); testing the deployed pipeline end to end on known inputs, including every preprocessing branch and any input router; and confirming that corrections reach every deployed copy.

### 4.5 Chronology of the audit

The original audit was exploratory, and several intermediate results were wrong, among them a perceptual hash whose errors canceled, a reflection check skipped by a programming error, a geometric estimate calibrated against a mis-specified null (Supplementary material S3), and a three-draw control experiment that more draws refuted. The revision corrected a further error in the development pipeline (the stochastic histopathology evaluation) and several errors in our first submission: superseded figures in the abstract, a comparison across two metrics, an inaccurate training description, a contradicted statement about gingivitis, a misdescribed reference for *R*^2^, a mismatched dataset citation, the missing generative AI statement, and the statement that the system had not been publicly deployed. Supplementary material S6 records each in order.

### 4.6 Limitations

This audit of one checkpoint from one training run is illustrative rather than generalizable. Duplication counts are lower bounds. The removal experiment has limited power. The analyses in Section 3.3 are secondary; the orientation analysis is exploratory and needs replication (Section 3.3). Total model exposure could not be audited (Section 4.1), and retraining on source-level partitions was not performed. Agreement between two annotations is not evidence that either is correct. Router results may include images that were present in its unretained training data. The clinical benchmark contains no healthy mouths, so screening specificity is unknown, and the histopathology set has 18 negatives from eight source images, none independent of training. No expert review of labels, external validation, clinical evaluation, or user study was performed.

### 4.7 Conclusion

A public dataset contains duplication across all three partitions that filename-based and fixed-alignment checks miss. In this system, that duplication did not detectably change aggregate mAP, whereas defects in the labels, the evaluation software, the thresholds, the router, and the class mapping changed what the evaluation reported or what the application displayed. Verifying partitions and labels, pinning the evaluation library, and validating the pipeline and its output end to end would each have caught a defect that model metrics did not.

## Data Availability

The datasets presented in this study can be found in online repositories. The audit code, pinned software environments, analysis plan and all audit outputs are available at https://github.com/VanVan120/Oral-Health-Computer-Vision-Model (tag v1.1-r1b; the analysis plan is ANALYSIS_PLAN_R1.md, commit 7ea8df2). The state of the repository at original submission is commit 9504bf16e9bd063f52aec357599ae3c2c20a6c92. The repository's history was rewritten on 23 September 2026 to remove development database files that held personal data; HISTORY_REWRITE.md in the repository maps these commit identifiers to the ones quoted before that date. Model weights are stored in the repository. Both image collections are public on Roboflow Universe under CC BY 4.0 [SEGP (University of Nottingham Malaysia), 2025; SEGP (University of Nottingham Malaysia), 2026; Oral Cancer, 2023].

## Supplementary material

Supplementary material S0 A guide to all supplementary files.

Supplementary material S1 124 test–training duplicate pairs found at fixed alignment.

Supplementary material S2 259 test–training pairs were found across the eight rotations and reflections, with the matching transform at thumbnail and full resolution and the originally published pairing.

Supplementary material S3 An exploratory sample of 200 test images scored by geometric (ORB/RANSAC) matching; its null distribution was mis-specified, so the analysis estimates no rate and is used in no analysis.

Supplementary material S4 Per-class duplication counts.

Supplementary material S5 The 96 histopathology validation images with an augmented copy of their source in training.

Supplementary material S6 The chronology of the audit, including every analysis revised after initial results.

Supplementary material S7 256 validation–training duplicate pairs, with their full-resolution verification.

Supplementary Tables S8.1–S8.9 Checkpoint provenance and training curve, per-class contrasts, image-level outcomes, router results, the pre-correction class-mapping path, library version and batch size, histopathology details, deviations from the analysis plan, and concordance between duplicated copies.
