# Supplementary material for: From benchmark to user: an end-to-end evaluation audit of an AI-based oral health screening prototype
Source: Front Big Data. 2026 Oct 28;9:1972023. doi: 10.3389/fdata.2026.1972023 (PMC13752011; doi:10.3389/fdata.2026.1972023)
Supplement: Supplementary file 1 [file Data_Sheet_1.zip › S6_audit_chronology.docx]

**Supplementary Material S6: Chronology of the audit**

*From benchmark to user: an end-to-end evaluation audit of an AI-based oral health screening prototype*

This record answers four questions: which analyses and thresholds were fixed before any metric was seen; which methods were changed after initial results, and why; which analyses remain exploratory; and whether every analysis attempted is documented. Times come from the repository history, the checkpoints’ metadata, the data platform and the original audit’s correction log (a methods appendix dated 13 August 2026). Where a time was not recorded we say so. Status codes: **F**, fixed before any metric was computed; **C**, changed after initial results; **E**, exploratory; **P**, pre-specified in the revision plan; **D**, deviation from that plan.

**Table S6.1.** Chronology of the audit.

| **Date** | **Stage** | **Event** | **Status** |
| --- | --- | --- | --- |
| ***Development of the system (before the audit)*** | | | |
| 6 Dec 2025 | Data | The only version of the clinical dataset generated on the platform (10,000 images, no augmentation). |  |
| 6 Dec 2025 | Model | Earlier detector checkpoint written: 50 epochs, library defaults, initialised from a checkpoint that was not retained. |  |
| 7 Dec 2025 | Code | First commit (f7e56f7): detector, histopathology and router weights, and a class-name dictionary whose order matches no checkpoint in the repository’s history. |  |
| 7 Dec 2025 | Deployment | Public demonstration of the application created on Hugging Face Spaces (a Docker Space serving the web application), with the same class-name dictionary. |  |
| 12 Dec 2025 | Model | Released detector checkpoint written (commit 122e026): tuned hyperparameters, stopped after epoch 48, weights from epoch 28 selected by validation fitness. |  |
| 3–5 Feb 2026 | Model | Histopathology dataset versions generated; histopathology model retrained and evaluated with the training transform applied to validation images (commit 492b247). |  |
| 1 Mar 2026 | Evaluation | Detector validation figures committed (commit 7c145fc); split, library version and batch size not recorded with them. |  |
| 24 Apr 2026 | Deployment | Last update of the public demonstration (commit fd32766), still with the pre-correction dictionary. |  |
| ***Original audit (August 2026; exploratory)*** | | | |
| Aug 2026 | Deployment | Class-mapping check: five of six conditions displayed under the wrong name. | E |
| Aug 2026 | Data | Both datasets re-downloaded at their pinned versions; identity checked against recorded split sizes. | F |
| Aug 2026 | Histopathology | Pooled re-split reproduced (435/109); 96 of 109 validation images have a training sibling. | E |
| Aug 2026 | Duplication | Fixed-alignment thumbnail rule; threshold 6.0 set from the distance distribution (no test image between RMS 3.92 and 10.01) before any metric was computed; 124 test images. The same set was recovered at thumbnail sizes from 8 × 8 to 64 × 64. | F |
| Aug 2026 | Duplication | Perceptual hash tried and discarded: 122 flags, but 5 of 12 inspected pairs were different images. | C |
| Aug 2026 | Duplication | Threshold validation that classed near-threshold pairs as distinct by mean pixel difference found invalid (it measured framing, not identity); all nine pairs were the same photograph re-cropped. | C |
| Aug 2026 | Histopathology | Leakage recomputed over 200 alternative orderings of the pooled index (73.4% to 93.6%). | E |
| 12 Aug 2026 | Deployment | A submission plan prepared that day recorded that the live system reported the wrong condition to users and scheduled a fix and redeployment. |  |
| 13 Aug 2026 | REV 1 | First inflation test removed the 124 images and compared with 3 random control sets; the conclusion (no inflation) was later found to rest on a contaminated comparison set. | C |
| 13 Aug 2026 | REV 2 | Orientation sweep added: 259 test images; the earlier comparison set contained 135 duplicates. | C |
| 13 Aug 2026 | REV 3 | A programming error had silently skipped the horizontal-flip arm; found by checking that each transform changes the image, and fixed. | C |
| 13 Aug 2026 | REV 4 | Geometric (ORB) estimate of 82.5% demoted to indicative: its null was mis-specified for a maximum over 60 candidates (now S3, exploratory). | C, E |
| 13 Aug 2026 | REV 5 | Framing changed from inflated results to a provenance defect. | C |
| 13 Aug 2026 | REV 6 | Apparent deficit of duplicated images traced to ulcer’s 3 instances; apparent per-class recall advantage traced to different operating points; claimed irreproducibility withdrawn after pinning ultralytics 8.3.231. | C |
| 13 Aug 2026 | REV 7 | Reproduction tolerance stated: 21 of 28 cells exact, 7 within 0.001. | C |
| 13 Aug 2026 | REV 8 | Control sets redrawn at the correct size (1,241 images). | C |
| 13 Aug 2026 | REV 9 | Control sets increased from 3 to 10; the 3-set result had been a small-sample artefact. | C |
| 13 Aug 2026 | Deployment | Class mapping corrected in the repository (commit 0b2546a, 15:55); as-submitted state (9504bf1, 16:02). The correction was not deployed to the public demonstration. |  |
| Aug 2026 | Submission | Manuscript submitted. Its abstract retained figures from the REV 1 analysis (an error, corrected in revision). |  |
| ***Errors in the original submission corrected in revision*** | | | |
| Sep 2026 | Submission | Abstract figures (0.7626 against 0.7670) came from the superseded 124-image analysis. |  |
| Sep 2026 | Submission | Former Section 3.3 compared a version effect on mAP@0.5:0.95 with sampling ranges on mAP@0.5. |  |
| Sep 2026 | Submission | Training described as 100 epochs with automatic batch sizing; the checkpoint records 200 epochs requested, 48 run, batch size 100. |  |
| Sep 2026 | Submission | Statement that a user with gingivitis had close to even odds of being told nothing was present, contradicted by the image-level analysis. |  |
| Sep 2026 | Submission | R² described as referenced to the training mean; it is referenced to the evaluation-set mean. |  |
| Sep 2026 | Submission | Clinical dataset cited to a different public project (about 4,162 images). |  |
| Sep 2026 | Submission | The statement that the system had not been publicly deployed was wrong: the public demonstration had been online since December 2025 and was still running the pre-correction code. |  |
| Sep 2026 | Submission | No Generative AI statement was included in the manuscript, although the use of generative AI (Claude, Anthropic) in the audit and in drafting had been declared in the submission form. |  |
| ***Revision (September 2026)*** | | | |
| 15 and 18 Sep 2026 | Review | Reports received; interactive review opened on 21 September. |  |
| 21 Sep 2026, 21:25 | Plan | Analysis plan committed and pushed (commit 7ea8df2) before any revision analysis was run. | P |
| 21 Sep 2026 | Data | Datasets re-downloaded at pinned versions; D reproduced exactly (259; fixed-alignment subset 124). | P, D1, D4 |
| 21 Sep 2026 | Evaluator | Cached evaluator reproduces the library’s validation exactly on four subsets. | P |
| 21 Sep 2026 | Statistics | Removal effect with cluster bootstrap; 10,000 stratified and 10,000 unstratified controls; minimum detectable effect. | P, D5 |
| 21 Sep 2026 | Validation | Validation partition with and without its 256 duplicates. Fitness was first computed with the plan’s formula (0.1·mAP@0.5 + 0.9·mAP@0.5:0.95), corrected on 22 September. | P |
| 21 Sep 2026 | Mechanism | Performance by partition; label concordance of duplicated pairs; recall against each annotation. | P |
| 21 Sep 2026 | Duplication | Photometric tier: 163 further candidates at full-resolution correlation ≥ 0.95, forming a continuum; tier dropped although it verified against the random-pair null. | P, D9 |
| 21 Sep 2026 | Version | Library-version effect decomposed: identical predictions; difference in average-precision integration. | P, E |
| 21 Sep 2026 | System | End-to-end run of 1,500 test images; pre-correction path simulated in full; router on three image sets; regression test. | P, D2, D3 |
| 21 Sep 2026 | System | A first reading of the code, that every benchmark image takes the sliced-inference branch, was reversed by the end-to-end run: none does, because every image is under 640 pixels high. | C |
| 21 Sep 2026 | Histopathology | Deployed checkpoint evaluated deterministically; augmented evaluation identified; filenames audited. | P |
| 21 Sep 2026 | Histopathology | A first conclusion that the originally reported metrics described a checkpoint that no longer exists was withdrawn: the deployed file is inferred to hold the best-epoch weights evaluated in the notebook. | C |
| 22 Sep 2026 | Concordance | Matching transform re-derived at full resolution (259 of 259 agree): the label disagreement is not a registration artefact. 24 pairs inspected side by side by the AI agent that ran the audit. Pairing compared with the originally published list (same training image for 222 of 259). | E, D8 |
| 22 Sep 2026 | Mechanism | Predictions on each copy compared with both annotations. The pooled comparison (+0.004, interval spanning zero) had first been read as no evidence of memorisation. Split by whether training augmentation could reproduce the pair’s orientation, the two groups differed in opposite directions (+0.074 and −0.081), and the pooled reading was withdrawn. | C, E, D8 |
| 22 Sep 2026 | System | The first draft of the results gave a with-router “No Issues Detected” rate of 26.9%, which combined images rejected by the router (22.9%) with analysed images with no finding (3.9%). Corrected, and outcomes split by condition (router rejection from 6.1% for calculus to 51.5% for ulcers). | C, D8 |
| 22 Sep 2026 | Statistics | Per-class contrasts rerun with the specified 10,000 resamples, reporting observed differences. A widened control stratum labelled caries-dominant in the first draft corrected to hypodontia-dominant; no number changed. | C, D6 |
| 22 Sep 2026 | Validation | Fitness recomputed as mAP@0.5:0.95, the library’s definition (shift −0.00473 rather than −0.00520). A draft statement that no checkpoint choice depended on validation contamination withdrawn as unsupported; the selection margin (0.00111) compared with the shift. All 256 validation pairs verified at full resolution. | C, D7, E |
| 22 Sep 2026 | Deployment | Public demonstration found still running the pre-correction code (commit fd32766); made private and taken offline the same day. |  |
| 22 Sep 2026 | Checking | Every reported number checked against the result files in a separate AI session: no numerical errors found; the labelling of exploratory analyses, several statements and the list of deviations corrected (D9, D10). | C, D9, D10 |
| 23 Sep 2026 | Repository | Two development database files holding personal data of test users removed from the repository’s history; 44 of 74 commit identifiers changed, tag names unchanged, and HISTORY_REWRITE.md records the mapping. Identifiers in this record are the current ones. |  |

No analysis attempted in the original audit or the revision is omitted from this record, to the authors’ knowledge. Figures produced in the original audit that were later superseded are not used in the revised manuscript except where it describes the error.
