# Supplementary material for: From benchmark to user: an end-to-end evaluation audit of an AI-based oral health screening prototype
Source: Front Big Data. 2026 Oct 28;9:1972023. doi: 10.3389/fdata.2026.1972023 (PMC13752011; doi:10.3389/fdata.2026.1972023)
Supplement: Supplementary file 1 [file Data_Sheet_1.zip › S8_Supplementary_Tables.docx]

**Supplementary Material S8: Supplementary tables**

*From benchmark to user: an end-to-end evaluation audit of an AI-based oral health screening prototype*

All detector metrics are from ultralytics 8.3.231 on CPU at batch size 1 unless stated. Every value is regenerated by audit/run_all.sh in the repository (tag v1.1-r1b), except the visual check in Table S8.9a and one robustness row in Table S8.9d, marked ¹; source files, in audit/results, are named in each table note. Analyses added after the results were seen are marked exploratory (Table S8.8, D8).

**Table S8.1.** Training records stored in the two detector checkpoints in the repository’s history.

| **Field** | **Released checkpoint** | **Earlier checkpoint (warm start)** |
| --- | --- | --- |
| Git object (LFS oid) / commit | 4de8714f…7aad0cc / 122e026 (12 Dec 2025) | eec5aba9…97946 / f7e56f7 (7 Dec 2025) |
| Date written / ultralytics version | 2025-12-12 16:33 / 8.3.231 | 2025-12-06 15:51 / 8.3.231 |
| Initialised from | an earlier best.pt in the project (the warm start) | best.pt of unknown origin (not retained) |
| Dataset directory | oral-diseases-1 (absolute path on a Linux machine) | oral-diseases-1 (relative path) |
| Epochs requested / run / patience | 200 / 48 (early stop) / 20 | 50 / 50 / 100 |
| Validation mAP@0.5 after epoch 1 / validation fitness of the retained weights | 0.722 / 0.38766 (epoch 28) | 0.444 / 0.3873 |
| Training time | 43 min | 46 min |
| Batch size / image size / device | 100 / 640 / one GPU | 32 / 640 / one GPU |
| Seed / deterministic | 0 / true | 0 / true |
| Optimiser / lr0 / lrf / momentum / weight decay | auto / 0.01555 / 0.0071 / 0.89158 / 0.00047 | auto / 0.01 / 0.01 / 0.937 / 0.0005 |
| Loss gains box / cls / dfl | 8.42592 / 0.72633 / 1.30086 | 7.5 / 0.5 / 1.5 |
| Augmentation: fliplr / flipud / degrees / mosaic / scale | 0.27883 / 0 / 0 / 0.98321 / 0.70338 | 0.5 / 0 / 0 / 1.0 / 0.5 |
| Run name (project) | yolov8m_evolved_final (oral_cancer_screening) | HPC_Run_Optimized (none) |
| Five best epochs by validation mAP@0.5:0.95 in the training log | 28 (0.38766), 8 (0.38655), 26 (0.38469), 25 (0.38459), 35 (0.38377); selection margin 0.00111 |  |
| Retained weights re-evaluated on validation, mAP@0.5:0.95: all 1,500 images / without the 256 duplicates | 0.38737 / 0.38264 (difference −0.00473, 4.3 times the selection margin) |  |

S10 and S14 in audit/results hold the complete 105-key training arguments. The released run’s non-default hyperparameters and run name suggest that they came from the library’s evolutionary tuning, which scores candidates on the validation partition; no record of the search was kept. The only version of the clinical dataset was generated on 6 December 2025. Fitness in ultralytics 8.3.231 is mAP@0.5:0.95 alone (Table S8.8, D7). The training log records validation scores computed during training; the re-evaluation uses the audit evaluator (CPU, batch size 1), hence 0.38737 rather than 0.38766 for the same weights. Only the retained weights and the warm start survive, so the de-duplicated score of the runner-up epoch cannot be computed. Source: S10, S14, S32, S53.

**Table S8.2.** Per-class contrasts between duplicated (D) and non-duplicated (ND) test images. Differences are D − ND with 95% cluster-bootstrap intervals.

| **Class** | **Instances D / ND** | **AP@0.5 D / ND** | **AP@0.5:0.95 D / ND** | **Δ recall at t = 0.282** | **Δ recall at deployed threshold** | **Δ precision at t = 0.282** | **Δ precision at deployed threshold** |
| --- | --- | --- | --- | --- | --- | --- | --- |
| Calculus | 455 / 1010 | 0.625 / 0.648 | 0.279 / 0.283 | +0.025 (−0.059, +0.109) | +0.022 (−0.061, +0.106) | −0.055 (−0.133, +0.023) | −0.054 (−0.130, +0.021) |
| Caries | 251 / 1443 | 0.751 / 0.838 | 0.387 / 0.444 | −0.037 (−0.122, +0.037) | −0.067 (−0.158, +0.011) | −0.057 (−0.156, +0.034) | −0.051 (−0.149, +0.040) |
| Gingivitis | 597 / 947 | 0.565 / 0.549 | 0.247 / 0.235 | +0.071 (−0.020, +0.166) | +0.068 (−0.025, +0.165) | −0.033 (−0.126, +0.057) | −0.033 (−0.125, +0.062) |
| Hypodontia | 144 / 190 | 0.799 / 0.746 | 0.382 / 0.332 | +0.027 (−0.092, +0.146) | +0.003 (−0.140, +0.147) | +0.011 (−0.110, +0.128) | +0.018 (−0.091, +0.124) |
| Tooth discoloration | 825 / 3328 | 0.812 / 0.891 | 0.497 / 0.588 | −0.048 (−0.105, +0.001) | −0.067 (−0.129, −0.012) | −0.069 (−0.132, −0.015) | −0.060 (−0.122, −0.007) |

t, the confidence at which the smoothed mean F1 curve of the full test split peaks. Deployed thresholds: calculus 0.25, caries 0.35, gingivitis 0.30, hypodontia 0.60, tooth discoloration 0.40. Ulcer is excluded (3 instances in D). Values are observed differences; intervals are percentiles of 10,000 cluster-bootstrap resamples, stratified by whether a cluster contains a duplicated image (Table S8.8, D6). The bootstrap mean differed from the observed difference by at most 0.0021. Estimation only: among ten recall and ten precision contrasts, an interval that excludes zero would not be surprising by chance. No intervals were computed for the per-class AP contrasts (Table S8.8, D10). Source: S32.

**Table S8.3.** Image-level outcomes on the 1,500 test images, through the application pipeline, as corrected, at its per-condition thresholds. For images annotated with each condition: how often the detector path reported it or reported nothing, and the four mutually exclusive outcomes once the router is in place (proportion, 95% Wilson interval). The four-way split of with-router outcomes is exploratory.

| **Condition** | **Images** | **Detector path: reported** | **Detector path: nothing reported** | **With router: rejected as not a valid image** | **With router: accepted, nothing reported (“No Issues Detected”)** | **With router: accepted, condition reported** | **With router: accepted, other conditions only** | **Reported when absent, detector path / with router** |
| --- | --- | --- | --- | --- | --- | --- | --- | --- |
| Ulcer | 274 | 0.715 (0.659–0.765) | 0.266 (0.218–0.322) | 0.515 (0.456–0.573) | 0.109 (0.078–0.152) | 0.365 (0.310–0.423) | 0.011 (0.004–0.032) | 0.000 / 0.000 |
| Tooth discoloration | 610 | 0.892 (0.865–0.914) | 0.007 (0.003–0.017) | 0.123 (0.099–0.151) | 0.002 (0.000–0.009) | 0.789 (0.754–0.819) | 0.087 (0.067–0.112) | 0.094 / 0.083 |
| Caries | 536 | 0.912 (0.885–0.933) | 0.013 (0.006–0.027) | 0.187 (0.156–0.222) | 0.011 (0.005–0.024) | 0.729 (0.690–0.765) | 0.073 (0.054–0.098) | 0.047 / 0.043 |
| Hypodontia | 184 | 0.685 (0.614–0.748) | 0.152 (0.107–0.211) | 0.223 (0.169–0.288) | 0.120 (0.080–0.174) | 0.516 (0.445–0.587) | 0.141 (0.098–0.199) | 0.002 / 0.001 |
| Calculus | 342 | 0.857 (0.816–0.890) | 0.006 (0.002–0.021) | 0.061 (0.041–0.092) | 0.006 (0.002–0.021) | 0.819 (0.774–0.856) | 0.114 (0.085–0.152) | 0.041 / 0.035 |
| Gingivitis | 297 | 0.785 (0.734–0.827) | 0.000 (0.000–0.013) | 0.081 (0.055–0.117) | 0.000 (0.000–0.013) | 0.717 (0.663–0.765) | 0.202 (0.160–0.251) | 0.038 / 0.037 |

Images: the number of test images annotated with the condition. The four with-router outcomes sum to 1 for each condition; only “accepted, nothing reported” is a detector miss. Images without a condition all carry at least one other condition; there are no healthy images. Over all 1,500 images: detector path, at least one finding 1,389 (92.6%), “No Issues Detected” 111 (7.4%); with the router, at least one finding 1,097 (73.1%; 95% CI 70.8–75.3%), “No Issues Detected” 59 (3.9%; 3.1–5.0%), rejected as not a valid image 344 (22.9%; 20.9–25.1%; 341 by the softmax threshold and 3 by the brightness gate). For the with-router outcomes, the pre-specified cluster-bootstrap intervals (10,000 resamples) differ from the Wilson intervals by at most 0.013, the largest differences arising where no event occurred; for the detector-path columns only Wilson intervals were computed (Table S8.8, D10). Source: S34, S45, S49.

**Table S8.4.** Router outcomes at the deployed softmax threshold (0.95), with 95% Wilson intervals.

| **Image set** | **n** | **Routed as intended** | **Rejected (“Unknown”)** | **Routed to the wrong pathway** | **Rejected by a quality gate** |
| --- | --- | --- | --- | --- | --- |
| Clinical test split (intended: clinical) | 1,500 | 1,156, 0.771 (0.749–0.791) | 344, 0.229 (0.209–0.251) | 0, 0.000 (0.000–0.003) | 3 (brightness < 40) |
| Histopathology images (intended: histopathology) | 544 | 410, 0.754 (0.716–0.788) | 134, 0.246 (0.212–0.284) | 0, 0.000 (0.000–0.007) | 0 |
| COCO128 (intended: rejection) | 128 | 117 rejected, 0.914 (0.853–0.951) | 117 | 11 accepted, 0.086 (0.049–0.147): 10 as clinical, 1 as histopathology | 3 (brightness < 40) |

The router is a two-class ResNet18 (clinical, histopathological) whose class names are fixed in code rather than stored in the checkpoint. Images are rejected when the top softmax probability is below 0.95 or when a quality gate fires (mean brightness < 40 or > 250, or standard deviation < 15); the last two gates never fired on these 2,172 images. The router’s training data were not retained, so overlap with these sets cannot be excluded. Source: S35, S46.

**Table S8.5.** The class-mapping defect at the user-facing layer: simulation of the pre-correction path (commit fd32766, the version the public demonstration was still running in September 2026) on the 1,500 test images.

| **Class index** | **Class in the model** | **Displayed before correction** | **Threshold applied before correction** | **Correct threshold** | **Detections** |
| --- | --- | --- | --- | --- | --- |
| 0 | calculus | Caries | 0.35 | 0.25 | 1,162 |
| 1 | caries | Calculus | 0.25 | 0.35 | 1,792 |
| 2 | gingivitis | Gingivitis (correct) | 0.30 | 0.30 | 1,178 |
| 3 | hypodontia | Tooth Discoloration | 0.40 | 0.60 | 301 |
| 4 | tooth_discolation | Ulcers | 0.75 | 0.40 | 1,962 |
| 5 | ulcer | Hypodontia | 0.60 | 0.75 | 413 |

Full pre-correction path (thresholds looked up by display name): 1,464 of 1,994 displayed findings (73.4%) carried a wrong name; 1,302 of 1,500 images (86.8%) showed at least one wrong name and 1,074 (71.6%) showed only wrong names. Mapping alone, with the correct thresholds: 1,582 of 2,106 findings (75.1%) wrong; 1,309 and 1,011 images. A finding is a distinct condition name displayed for an image. The permutation is one transposition (0↔1) and one three-cycle (3→4→5→3). The dictionary matched no checkpoint in the repository’s history from its first commit (f7e56f7, 7 December 2025) until its removal (0b2546a, 13 August 2026). Detections: box-level counts in the simulation. Source: S34, S46, S15.

**Table S8.6.** Evaluation-library version and batch size, same checkpoint and test images, CPU.

| **Setting** | **mAP@0.5** | **mAP@0.5:0.95** | **Precision** | **Recall** |
| --- | --- | --- | --- | --- |
| 8.3.231, batch 16 (reproduces the development values) | 0.7710 | 0.4010 | 0.7452 | 0.7284 |
| 8.3.231, batch 1 (the evaluator used throughout) | 0.7709 | 0.4009 | 0.7454 | 0.7312 |
| 8.4.118, batch 16 | 0.7668 | 0.3892 | 0.7452 | 0.7284 |
| 8.4.118, batch 1 | 0.7668 | 0.3891 | 0.7454 | 0.7312 |
| Version effect, batch 16 (8.4.118 − 8.3.231) | −0.0042 | −0.0118 | 0.0000 | 0.0000 |
| Removal effect (ND − full), 8.3.231, batch 1 | +0.0010 | +0.0011 |  |  |
| Removal effect (ND − full), 8.4.118, batch 1 | +0.0004 | +0.0008 |  |  |

Across the 1,500 images, the 61,159 predictions and their match statistics (true-positive matrix, confidences, classes, targets) are bit-identical between versions; only the average-precision integration differs. Version 8.3.231 appends a sentinel at recall 1.0 with precision 0, so 101-point interpolation credits a linear precision ramp from the highest recall reached to recall 1.0; version 8.4.118 inserts a point at (highest recall, 0), so recall beyond that point contributes nothing, as in the COCO evaluation. Batch size changes letterbox padding under rectangular batching; at batch size 16 the per-class values reported during development are reproduced to within 0.001, and batch size 1 changes per-class recall by up to 0.009 (hypodontia, 0.7395 at batch size 16 against 0.7485 at batch size 1). The development batch size itself was not recorded. Source: S28, S29, S33, S44.

**Table S8.7.** Histopathology: sources of the negative images, repeated augmented evaluations, and the training partition.

| **Quantity** | **Value** |
| --- | --- |
| TVNT-negative images / distinct source images | 18 / 8 (OSCC_400x_159 ×3, 222, 291 ×3, 317, 322 ×3, 349, 366 ×3, 493 ×3) |
| Validation negatives / with their source image in training | 4 / 4 (sources 291, 322, 493) |
| Distinct source images: all 544 images / validation partition (109 images) | 228 / 94; the classifier called every source positive, as it did every image |
| Validation images without a training sibling | 13, all TVNT-positive (sensitivity 1.000, 0.753–1.000; other metrics undefined) |
| Validation ROC-AUC, five evaluations with the inherited training transform | 0.767, 0.788, 0.652, 0.769, 0.605 (confusion matrix unchanged in all five) |
| Training partition (n = 435): confusion matrix TN / FP / FN / TP | 0 / 14 / 0 / 421 |
| Training partition: sensitivity / specificity / balanced accuracy | 1.000 (0.991–1.000) / 0.000 (0.000–0.232) / 0.500 |
| Training partition: ROC-AUC / AP positive / AP negative | 0.955 (0.905–0.992) / 0.998 / 0.682 |
| Relation of the deployed file to the evaluated checkpoint | model_a.pth is inferred to hold the best-epoch weights evaluated in the notebook (consecutive execution order; identical confusion matrices on both partitions) |

The validation subset inherited the training transform (random horizontal and vertical flips, rotation up to 15° and colour jitter) because the pooled dataset object was created with it and split into subsets; the deterministic evaluation uses resizing and normalisation only. Source: S36, S37, S42.

**Table S8.8.** Deviations from the pre-specified analysis plan (ANALYSIS_PLAN_R1.md, commit 7ea8df2).

| **ID** | **Deviation** | **Reason** |
| --- | --- | --- |
| D1 | The check that the fixed-alignment subset reproduces S1 was implemented as images below threshold under the identity transform (124), not images whose best transform is the identity (63) | The analysis specification’s wording was ambiguous; the published S0 README defines the subset this way |
| D2 | The pre-correction path was simulated in full (old dictionary and thresholds looked up by display name) as the primary estimand; the mapping-only estimand is secondary | The thresholds were keyed by display name, so the mapping alone does not reproduce what users would have seen |
| D3 | Quality-gate rejections are counted separately from softmax rejections | An image rejected by a gate is never classified |
| D4 | Thumbnails are decoded without JPEG draft mode | Draft-mode decoding pads JPEG blocks asymmetrically and lost 31 of the 259 pairs (12%), all of them reflected or rotated; the published S2 is reproduced exactly without it |
| D5 | Stratified control sets are drawn greedily without reusing an image; two strata were widened to the adjacent bin | Two strata could not be filled from their own bin: caries-dominant images with 16 or more instances (4 needed, none available) and hypodontia-dominant images with one instance (26 needed, 25 available) |
| D6 | Per-class contrasts were first computed with 2,000 resamples and reported the bootstrap mean as the point estimate; they were rerun with the specified 10,000 resamples, reporting the observed difference | The first run did not follow the plan. The bootstrap mean differed from the observed difference by at most 0.0021, and no conclusion changed |
| D7 | Validation fitness is reported as mAP@0.5:0.95, not as 0.1·mAP@0.5 + 0.9·mAP@0.5:0.95 as the plan stated | The plan’s formula was wrong for this library version: ultralytics 8.3.231 selects checkpoints on mAP@0.5:0.95 alone, as the checkpoint’s stored fitness confirms |
| D8 | Added after the results and therefore exploratory: the full-resolution transform check for label concordance; the comparison of predictions with both annotations, overall, by transform and by whether training augmentation could reproduce the pair’s orientation; the visual check of 24 pairs; the four-way split of router outcomes; and the full-resolution verification of validation pairs | To test whether the label disagreement was a registration artefact; to extend the pre-specified recall comparison against each annotation to box-level agreement, and to separate pairs in which memorisation of the paired training copy was and was not possible; and to correct a reporting error that combined rejected and analysed images |
| D9 | The photometric tier (plan section 3.8) was dropped, although its candidates verified against the random-pair null | The 280 candidates formed a continuum with no natural threshold (163 verified at full-resolution r ≥ 0.95, 117 not), and the tier did not reproduce at the size the plan stated (422 images rather than 322), so any tier would have depended on a threshold chosen after the fact |
| D10 | Not computed or not reported: 95% intervals for the partition-level mAPs, the recall of predictions against each annotation, the training copies’ mAP@0.5, the validation de-duplication shift and the per-class AP contrasts (plan 3.1 and 3.2); cluster-bootstrap intervals for the detector-path and “reported when absent” image-level proportions, of which only the detector-path proportions have Wilson intervals (plan 3.4); and histopathology metrics at source level beyond the counts in Table S8.7 (plan 3.7) | Oversights in the run and in reporting. At source level the classifier result is unchanged, because every image, and therefore every source, was called positive |

Deviations D1 to D8 are recorded, with dates, in the plan’s DEVIATIONS section in the repository (tag v1.1-r1b); D9 and D10 were identified in checking this revision and are recorded here. Source: ANALYSIS_PLAN_R1.md, S40.

**Table S8.9a.** Duplicated test images and their training copies: registration, pairing and verification (259 test–training pairs unless stated). Exploratory.

| **Check** | **Result** |
| --- | --- |
| Matching transform, 32 × 32 thumbnails against 256 × 256 full resolution | Same in 259 of 259 pairs (identity 59, horizontal flip 80, vertical flip 77, 180° rotation 43); none resolves to a 90° rotation or a transposition |
| Margin of the best transform: RMS of the runner-up divided by RMS of the best | Minimum 3.64, median 25.3; no pair below 2, so the agreement above is a consistency check rather than independent confirmation |
| Pixel dimensions | Identical in 258 pairs; the remaining pair is the same picture at two scales (aspect ratios within 0.5%) |
| Best achievable concordance over the four transforms, per pair (oracle) | Mean class-aware F1 0.669 at the chosen transform and 0.670 at the best; 2 pairs would gain |
| Pairing compared with the originally published list (S2 of the original submission) | Same training image in 222 pairs and same transform in 225; the set of 259 test images is identical. Where the training image differs, the two candidates are usually almost equally close (median relative RMS difference 3.8%). Under the original pairing, 21 pairs would fall in the other orientation group of Table S8.9c |
| Concordance under the originally published pairing | Class-aware F1 0.685; identical label sets 22.8% |
| Full-resolution verification (Pearson r ≥ 0.95 after standardisation, best of eight transforms) | Test pairs 259 of 259 (minimum r 0.960); validation pairs 256 of 256 (minimum r 0.982); random-pair null median r 0.20 |
| Visual check of 24 pairs, stratified by transform (3 sheets of 8, seed 20260921) | Same photograph in 24 of 24; labels differ in 20. Commonest difference: a whole condition annotated on one copy only (at least 10 pairs); a lesion given a different class (2 pairs). The four agreeing pairs have one or two boxes each and are the four the computed flag calls identical |

The transform maps the test image onto its training copy, and its inverse carries the training copy’s boxes into the test image’s frame. The pairing used throughout the revision is S2 of this Supplementary Material. The 24 pairs were inspected by the AI agent that ran the audit (Claude Opus 5), not by an author; the sheets are not distributed because they contain dataset images. Source: S47, S48, S50, S51, S52.

**Table S8.9b.** Agreement between the test annotation and the training copy’s annotation of the same photograph, by the transform relating them.

| **Transform** | **Pairs** | **Boxes, test / training copy** | **Box F1, class-agnostic** | **Box F1, class-aware** | **Identical label sets** |
| --- | --- | --- | --- | --- | --- |
| Identity | 59 | 595 / 551 | 0.749 | 0.745 | 17 (28.8%) |
| Horizontal flip | 80 | 650 / 668 | 0.690 | 0.659 | 19 (23.8%) |
| Vertical flip | 77 | 611 / 592 | 0.623 | 0.610 | 14 (18.2%) |
| 180° rotation | 43 | 419 / 419 | 0.644 | 0.628 | 7 (16.3%) |
| All pairs | 259 | 2,275 / 2,230 | 0.679 | 0.662 | 57 (22.0%) |

Greedy matching at intersection over union ≥ 0.5 after mapping the training copy’s boxes into the test image’s frame; F1 pooled over pairs. Class-aware precision and recall over all pairs: 0.669 and 0.655. Source: S47.

**Table S8.9c.** The detector’s predictions on each copy compared with both annotations (class-aware box F1 at t = 0.282), by transform. Exploratory.

| **Pairs related by** | **Pairs** | **(i) Predictions on test image vs on training copy** | **(ii) Test annotation vs training-copy annotation** | **(iii) Predictions on test image vs test annotation** | **(iv) Predictions on test image vs training-copy annotation** | **(v) Predictions on training copy vs its annotation** | **(iv) − (iii), 95% CI** |
| --- | --- | --- | --- | --- | --- | --- | --- |
| Identity | 59 | 0.905 | 0.745 | 0.683 | 0.752 | 0.793 | +0.068 (+0.009, +0.125) |
| Horizontal flip | 80 | 0.842 | 0.659 | 0.681 | 0.759 | 0.806 | +0.078 (+0.024, +0.132) |
| Orientation seen in training, pooled | 139 | 0.870 | 0.699 | 0.682 | 0.756 | 0.800 | +0.074 (+0.033, +0.113) |
| Vertical flip | 77 | 0.670 | 0.610 | 0.668 | 0.571 | 0.773 | −0.097 (−0.163, −0.036) |
| 180° rotation | 43 | 0.715 | 0.628 | 0.691 | 0.633 | 0.841 | −0.058 (−0.138, +0.011) |
| Orientation not seen in training, pooled | 120 | 0.688 | 0.617 | 0.677 | 0.596 | 0.801 | −0.081 (−0.135, −0.032) |
| All pairs | 259 | 0.788 | 0.662 | 0.680 | 0.684 | 0.801 | +0.004 (−0.032, +0.040) |
| Difference between the pooled groups (seen minus not seen) | | | | | | | +0.155 (+0.095, +0.218) |

Training augmentation used horizontal flips (probability 0.28) but no vertical flips or rotations, so only identical and horizontally flipped copies could have been presented to the detector in the test image’s orientation. The grouping was chosen after the per-transform results were seen. A positive (iv) − (iii) means predictions on the test image agree better with the training copy’s annotation than with the test image’s own. Overall 95% CIs: (i) 0.759–0.816, (ii) 0.623–0.700, (iii) 0.645–0.713, (iv) 0.646–0.720, (v) 0.769–0.831. Intervals: cluster bootstrap over the 214 clusters that contain a duplicated image, 10,000 resamples. Source: S47, S48, S52.

**Table S8.9d.** Sensitivity of the comparison in Table S8.9c. Values are (iv) − (iii) unless stated.

| **Variation** | **All pairs** | **Orientation seen in training** | **Orientation not seen in training** |
| --- | --- | --- | --- |
| Confidence threshold 0.05 | −0.007 | +0.026 | −0.045 |
| Confidence threshold 0.10 | −0.004 | +0.044 | −0.061 |
| Confidence threshold 0.20 | −0.002 | +0.061 | −0.078 |
| Confidence threshold 0.282 (t) | +0.004 | +0.074 | −0.081 |
| Confidence threshold 0.35 | +0.005 | +0.072 | −0.078 |
| Confidence threshold 0.45 | +0.012 | +0.083 | −0.076 |
| Confidence threshold 0.60 | +0.016 | +0.079 | −0.061 |
| Pooled weighting: per pair (macro) / one pair per distinct training copy | +0.000 / +0.013 |  |  |
| Matching within each class rather than class-agnostic then filtered | +0.004 |  |  |
| Excluding the 68 pairs whose test image also has a different training copy at fixed alignment (S1) ¹ | −0.007 (191 pairs) | +0.064 (102) | −0.084 (89) |
| (i) and (ii) in the 53 pairs with equal box counts in both comparisons | (i) 0.877, (ii) 0.869 |  |  |

The pooled difference changes sign across thresholds; the two groups keep opposite signs at all seven. The 259 pairs involve 224 distinct training copies (29 used twice and 3 three times). Over all pairs, (i) exceeds (ii) by 0.126, but by only 0.008 in the pairs where both comparisons have equal numbers of boxes on each side, so most of that gap reflects agreement about how many boxes there are rather than where they are. ¹ A second training copy in the test image’s own orientation would let the detector memorise that copy’s labels even when the nearest copy is flipped or rotated; excluding such pairs leaves both groups’ signs unchanged. This row was computed for this table from the per-pair counts in S48 and the pairs in S1, and is not produced by audit/run_all.sh. Source: S52; S48 and S1 for ¹.
